# Rapid and automated interpretation of CRISPR-Cas13-based lateral flow assay test results using machine learning†

**DOI:** 10.1039/d4sd00314d

**Published:** 2024-12-26

**Authors:** Mengyuan Xue, Diego H. Gonzalez, Emmanuel Osikpa, Xue Gao, Peter B. Lillehoj

**Affiliations:** a Department of Bioengineering, Rice University Houston TX 77030 USA lillehoj@rice.edu; b Department of Chemical and Biomolecular Engineering, Rice University Houston TX 77005 USA; c Department of Chemical and Biomolecular Engineering, University of Pennsylvania Philadelphia PA 19104 USA; d Department of Bioengineering, University of Pennsylvania Philadelphia PA 19104 USA; e Center for Precision Engineering for Health, University of Pennsylvania Philadelphia PA 19104 USA; f Department of Mechanical Engineering, Rice University Houston TX 77005 USA

## Abstract

CRISPR-Cas-based lateral flow assays (LFAs) have emerged as a promising diagnostic tool for ultrasensitive detection of nucleic acids, offering improved speed, simplicity and cost-effectiveness compared to polymerase chain reaction (PCR)-based assays. However, visual interpretation of CRISPR-Cas-based LFA test results is prone to human error, potentially leading to false-positive or false-negative outcomes when analyzing test/control lines. To address this limitation, we have developed two neural network models: one based on a fully convolutional neural network and the other on a lightweight mobile-optimized neural network for automated interpretation of CRISPR-Cas-based LFA test results. To demonstrate proof of concept, these models were applied to interpret results from a CRISPR-Cas13-based LFA for the detection of the SARS-CoV-2 N gene, a key marker for COVID-19 infection. The models were trained, evaluated, and validated using smartphone-captured images of LFA devices in various orientations with different backgrounds, lighting conditions, and image qualities. A total of 3146 images (1569 negative, 1577 positive) captured using an iPhone 13 or Samsung Galaxy A52 Android smartphone were analyzed using the trained models, which classified the LFA results within 0.2 s with 96.5% accuracy compared to the ground truth. These results demonstrate the potential of machine learning to accurately interpret test results of CRISPR-Cas-based LFAs using smartphone-captured images in real-world settings, enabling the practical use of CRISPR-Cas-based diagnostic tools for self- and at-home testing.

## Abstract 2

We present two machine learning models for rapid and automated interpretation of smartphone-captured images of a CRISPR-Cas13-based lateral flow assay for the detection of the SARS-CoV-2 N gene, which classified the test results with 96.5% accuracy.

## Graphical abstract

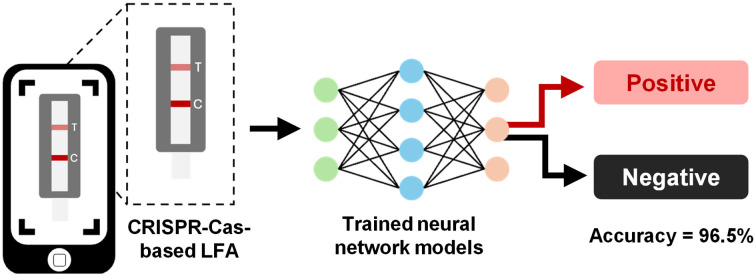

## 1. Introduction

Lateral flow assays (LFAs) are important diagnostic devices that offer a rapid, inexpensive, and straightforward method for detecting analytes in biofluid samples. Due to their portability, low cost, and ease of use, lateral flow-based serological tests are commonly employed for rapid diagnostic testing, particularly in detecting infectious diseases, such as malaria, HIV infection, and SARS-CoV-2, making them one of the most widely used diagnostic tools globally.^1,2^ Recently, CRISPR-Cas-based LFAs have emerged as a promising diagnostic platform for ultrasensitive nucleic acid detection,^3–7^ providing a faster and simpler alternative to polymerase chain reaction (PCR)-based assays, with minimal equipment required for field-based diagnostic testing. Most LFAs employ a colorimetric readout, where visible lines on the test strip indicate a positive or negative result, allowing users to analyze the results without specialized instrumentation or electricity. Despite these advantages, visual interpretation of LFA test results is challenging due to variability in the appearance of the test/control lines (*e.g.*, faint or nonuniform lines), individual differences in vision, and human error, which can result in false-positive or false-negative test results.^8,9^

To address these issues, electronic readers have been developed to enhance the sensitivity, accuracy, and reproducibility of LFAs.^10^ While effective, these devices are expensive (over $1000) and add extra steps to the testing process, limiting their use for at-home testing or in resource-limited settings. Recently, machine learning (ML) algorithms have been used for automated interpretation of LFA results, enhancing accuracy and reproducibility without the need for instrumentation.^11–22^ However, these methods often rely on custom hardware (*e.g.*, lateral flow strip holders/cradles, smartphone attachments), precise camera/lateral flow strip positioning and ideal lighting conditions, which limit their use in real-world environments.

In this work, we present a unique approach for rapid and automated interpretation of CRISPR-Cas-based LFA test results using ML. We employ a fully convolutional neural network (U-Net) and a lightweight mobile-optimized neural network (MnUV3) to accurately detect and classify test/control lines on CRISPR-Cas-based LFA images as a positive or negative test result. While lightweight neural networks have been used for ophthalmic image analysis,^23^ hair segmentation,^24^ and spinal cord segmentation^25^ applications, this is the first application for the interpretation of LFA test results. Unlike the previous ML-based methods, our approach accounts for variations in lighting, backgrounds, and image resolution without requiring manual image cropping or specific camera placement, all of which simplify the testing process for real-world environments. We demonstrate proof of concept by applying these models to analyze images of a CRISPR-Cas13-based LFA for the detection of the SARS-CoV-2 N gene. The trained models were validated using 3146 images (1569 negative, 1577 positive) of tested LFA devices captured using an iPhone 13 or Samsung Galaxy A52 smartphone, which revealed their ability to rapidly (within 0.2 s) classify them as positive or negative with 96.5% accuracy. We envision that these models can be applied to a wide range of CRISPR-Cas-based LFAs, enabling the detection of nucleic acid targets for various pathogens and genetic diseases, thus expanding the utility of this platform for rapid, at-home diagnostic testing.

## 2. Experimental

### 2.1 Expression and purification of LwaCas13a

The methods and procedures for the expression and purification of LwaCas13a were identical to those used in our prior work.^26^*E. coli* BL21(DE3) competent cells were electroporated with 20 ng of a protein expression vector and grown on a lysogeny broth (LB)-agar plate (Fisher Scientific) overnight. A single colony was picked and inoculated in 100 mL of starter culture and grown overnight, and 25 mL was transferred to a 1 L flask of LB with ampicillin (GoldBio) to a final concentration of 100 μg mL^−1^. The cultures were then grown at 37 °C and 220 rpm until the OD600 reached 0.4–0.6. The culture was cooled on ice, and the shaker was lowered to 18 °C. Cells were induced with 500 μL of 1 M isopropylthio-β-galactoside (IPTG) (GoldBio) for 16 h at 160 rpm. Cells were collected by centrifugation (Beckman Coulter Avanti J-E) at 4000*g* for 40 min and stored at −80 °C or used immediately. Five buffers were prepared: buffer A (20 mM Tris-HCl pH 7.5, 1 M NaCl with 3 mM 2-mercaptoethanol [BME]), buffer A1 (20 mM Tris-HCl pH 7.5, 1 M NaCl, 10 mM imidazole, 3 mM BME), buffer A2 (20mM Tris-HCl pH 7.5, 1 M NaCl, 30 mM imidazole, 3 mM BME), buffer B (20 mM Tris-HCl pH 7.5, 150 mM NaCl, 300 mM imidazole, 3 mM BME), buffer C (20 mM Tris-HCl pH 7.5, 150 mM NaCl with 1 mM DTT), and buffer D (20 mM Tris-HCl pH 7.5, 1 M NaCl with 1 mM dithiothreitol [DTT]). For every 10 mL of cells, 30 mL of buffer A and an EDTA-free protease inhibitor tablet (Thermo Scientific, Cat. A32965) were added. Cells were mixed until homogeneous by either rotation or vortex. Cells were then sonicated (Branson SFX550) with a 3 s on/6 s off interval for 2 min and 30 s total, with a 60% input. Meanwhile, Ni-NTA agarose beads (Qiagen, Germany) were then washed in buffer A and added to a gravity flow column (Marvelgent Biosciences). After sonication, the disrupted cell lysate was centrifuged (Beckman Coulter Optima L-90K ultracentrifuge) at 18 000*g* for 45 min at 4 °C. Once complete, the supernatant was transferred to the washed Ni-NTA beads. After the first flowthrough, ∼20 mL of buffer A was used to wash the column, then 10 mL of buffer A1, then 10 mL of buffer A2. The column was then placed over a collection tube and 10 mL of buffer B was used to elute the protein. Lab-purified SUMO-protease was then used to remove the SUMO-tag, with 200 μL of SUMO protease for every 10 mL of elution. All fractions were collected and run on an SDS-Page gel for verification. The collected, cleaved elute was then loaded onto a MonoS cation exchange column (Cytiva) to then be eluted by FPLC (AKTA PURE, GE Healthcare). The elution was done over a salt gradient of buffer C to buffer D (150 mM NaCl to 1 M NaCl). Pooled fractions of the protein from the FPLC were concentrated into a storage buffer (50 mM Tris-HCl, 600 mM NaCl, 5% glycerol, and 2 mM DTT, pH 7.5), and concentrated to 1.0–1.5 mg mL^−1^ using an Amicon Ultra centrifugal filter unit with 100 kDa cutoff (Millipore, Cat. UFC910024). The final elute was aliquoted and flash-frozen in liquid nitrogen before being stored at −80 °C.

### 2.2 Synthesis of the SARS-CoV-2 N gene target, crRNA, and LFA reporter

The methods and procedures for synthesizing the SARS-CoV-2 N gene target, crRNA, and LFA reporter were similar to those used in our prior work^26^ (Table S1†). The RNA target and crRNA were obtained by *in vitro* transcription (IVT) using a HiScribe T7 Quick High Yield RNA Synthesis Kit (New England Biolabs, E2050S). IVT templates for the SARS-CoV-2 N gene target were PCR-amplified from gBlock (IDT) containing a T7 promoter sequence using Pr18 (GAAATTAATACGACTCACTATAGGG) as the forward primer and Pr19 (CGCGCCCCACTGCGTTCTCC) as the reverse primer. PCR products were gel-purified and eluted with nuclease-free water. The concentration and purity of the templates were measured using a NanoDrop spectrophotometer (Thermo Scientific). At least 1 pmol of DNA was added to each IVT reaction.

IVT templates for the SARS-CoV-2 N gene crRNA were obtained by annealing the top primer (IDT synthesized oligomer of top strand) with the bottom primer (IDT synthesized oligomer of bottom strand). Briefly, 10 μM top and bottom primers were added to a 10 μL reaction solution containing 1 μL of 10× standard Taq buffer (New England Biolabs, B9014S). Annealing was performed in a thermocycler by heating the oligonucleotides to 95 °C and cooling them down to room temperature at 1 °C min^−1^. The 10 μL annealing reactions were directly used as IVT templates.

A 40 μL IVT reaction was performed by mixing the DNA template with 2.5 mM nucleoside triphosphates (New England Biolabs), 2 μL of T7 polymerase (New England Biolabs), and 1 U μL^−1^ Murine RNase Inhibitor (New England Biolabs, M0314L), followed by incubation at 37 °C for 4 h. The IVT products were treated with DNase I and purified by urea-PAGE gel electrophoresis followed by acid phenol–chloroform extraction. Briefly, bands were excised, crushed, and suspended in five volumes of 0.3 M NaOAc (pH 5.2, Thermo Scientific, R1181), and subjected to four repeated cycles of 15 min freezing at −80 °C and quick thawing at room temperature. The elute was filtered and mixed with an equal volume of phenol : chloroform : isoamyl alcohol (125 : 24 : 1, pH 4.5; Sigma-Aldrich, P1944), then centrifuged at 13 000*g* and 4 °C for 10 min. The upper aqueous phase was re-extracted with an equal volume of chloroform : isoamyl alcohol (24 : 1; Sigma-Aldrich, C0549) twice. For every 400 μL of washed upper phase, 1 μL of RNA-grade glycogen (Thermo Scientific, R0551) was added as an inert carrier of RNAs, and then mixed thoroughly with 400 μL of isopropanol to precipitate at −20 °C for 1 h. The resulting RNA pellet was washed with 70% ice-cold ethanol twice, air-dried, and redissolved in nuclease-free water. The RNA concentration and purity were measured using NanoDrop and the identity was confirmed by denaturing gel electrophoresis.

### 2.3 Design and fabrication of the LFA device

The LFA device consists of a HybriDetect lateral flow test strip (Milenia Biotec) which is housed inside a custom cassette (Fig. S1†). The cassette was designed using Fusion360 software (Autodesk) and fabricated from PLA filament (OVERTURE) using an Original Prusa MINI+ 3D printer. The bottom of the test strip extends outside of the cassette allowing the sample pad to be dipped into a tube containing the Cas reaction mixture. “T” and “C” labels are located next to the test result window to indicate the location of the test and control lines, respectively.

### 2.4 Detection of the SARS-CoV-2 N gene using a CRISPR-Cas13a-based LFA

2 μL of the target was added to a 1.5 mL tube containing the Cas13 detection reaction components: 2 μL of LwaCas13a (63.3 μg mL^−1^), 1 μL of RNase inhibitor (40 U μL^−1^, Lucigen), 0.6 μL of T7 RNA polymerase (50 U μL^−1^, Lucigen), 1 μL of crRNA for the SARS-CoV-2 N gene (10 ng μL^−1^), 1 μL of MgCl_2_ (120 mM), 0.8 μL of ribonucleoside triphosphate (RTP) mixture (25 mM), 2 μL of cleavage buffer (400 mM Tris pH 7.4) and 1 μL of biotin–FAM ssRNA reporter (20 μM, IDT). The Cas13 detection reaction mixture was incubated at 37 °C for 30 min. 80 μL of HybriDetect assay buffer (Milenia Biotec) was added to the Cas reaction tube and mixed thoroughly. A HybriDetect test strip was dipped into the reaction tube, and after 5 min, images of the LFA device were captured using a smartphone.

### 2.5 Design of the neural network model architecture

The neural network models consist of a segmentation module and a classification module. The segmentation module automatically detects the test/control line(s) in the LFA device images and converts them into black and white binary label images (termed binary labels) to identify the lines' location. We evaluated two different neural network architectures, U-Net and MnUV3, for the segmentation module. The binary labels are then fed into the classification module which classifies them as a positive or negative test result.

#### 2.5.1 Segmentation module

##### U-Net

U-Net is a network architecture initially proposed by Ronneberger, Fischer, and Brox for cell nuclei segmentation.^27^ U-Net follows an encoder–decoder network and is a fully convolutional network that utilizes a large number of trainable parameters to achieve accurate segmentation results. Additionally, it maintains the image size from the input to the output. The contracting path of the network has repeated application of unpadded convolutions, a ReLU operation, and a max pooling operation for down-sampling. The expansive path up-samples the feature map and eventually maps the feature vector to a binary output with the same first two dimensions as the input. Due to its fully convolutional feature, the U-Net architecture has 30 M parameters, limiting its use on mobile devices. In this work, we modified the input dimension so that three-channel (R, G, B) images can be directly fed into the network.

##### MnUV3

We created MnUV3, a lightweight network, in an attempt to reduce the computational load of the model. MnUV3 combines features from the MobilenetV3^28^ and U-Net architectures, making it suitable for implementation on mobile devices. The primary modifications involved changing the building blocks of the U-Net architecture. We kept the symmetric U-Net structure but removed the fully convolutional blocks to reduce the parameter count. MnUV3 integrates MobilenetV3 in the encoder section with U-Net in the decoder section. The encoder of the network consists of multiple MobilenetV3 blocks, which were built with subblocks for expansion convolution, depth-wise convolution, squeeze-and-excite module, and a pointwise convolution. MobilenetV3 blocks added a new h-swish function that is an approximation function with ReLU instead of sigmoid to significantly reduce the calculation time. The decoder of the network consists of the original expansive path from the U-Net architecture and upsamples the feature map multiple times to reach the same dimension as the input. The MnUV3 architecture has 18 M parameters, making it suitable for mobile applications.

#### 2.5.2 Classification module

A simple classification module (named ClassNet) was built consisting of a series of convolution and max pooling layers, followed by a softmax classifier that maps the features to an output of continuous probability scores ranging from 0 to 1. We applied a convolution layer followed by a ReLU activation function; then, we applied a max pooling layer with a kernel size of 2 × 2. The operation is applied four times, and the number of output channels in the convolution layer doubles the number of input channels each time the operation is applied. We then integrated a dropout layer with a dropout probability of 0.1 that randomly drops nodes within the network.

### 2.6 Data generation, model training, and model validation

The data used to train, evaluate and validate the neural network models consisted of LFA device photos, cropped images of LFA device photos and binary labels generated from the cropped images.

#### LFA device photos

**Fig. 1:**
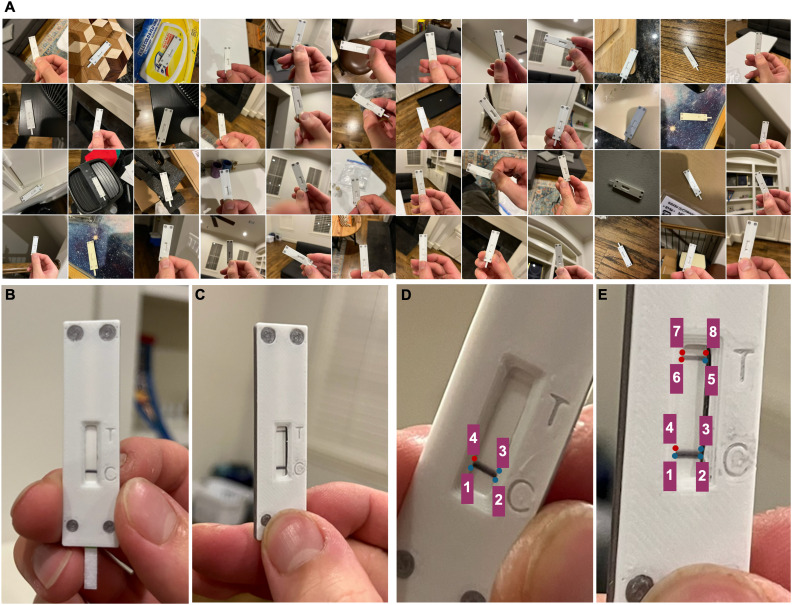
Smartphone-captured photos of tested LFA devices used to train and validate the ML models. (A) Collage of LFA device photos captured in real-world environments. Representative photos of an LFA device tested on a (B) negative sample and (C) positive sample. The process for manually annotating photos of LFA devices tested on a (D) negative sample and (E) positive sample.

Photos of tested LFA devices were captured using an iPhone 13 or Samsung Galaxy A52 Android smartphone. LFA devices were tested using 28 samples spiked with varying concentrations of the SARS-CoV-2 N gene, of which 14 were classified as true positives and 14 were classified as true negatives. Photos of LFA devices were captured using different phone orientations and LFA device-to-phone positions on various objects and surfaces, including a sofa, chair, coffee table, kitchen countertop, carpet, and bed, under various lighting conditions. This resulted in a highly diverse dataset that included images with suboptimal characteristics, such as overexposure, underexposure, and blurriness. The resulting dataset consisted of 637 unique LFA device photos (Fig. 1A), consisting of 294 photos tested on negative samples (Fig. 1B) and 343 photos tested on positive samples (Fig. 1C).

#### Human-annotated labels

The location of the test/control line(s) in the LFA device photos was manually annotated by finding the point coordinates and transforming the pixels into binary labels with 0s and 1s using MATLAB. For LFA device photos with only the control line appearing, three pairs of point coordinates were recorded (blue dotted points 1–3) and a fourth coordinate (red dotted point 4) was generated to form a parallelogram representing the line (Fig. 1D). For LFA device photos with both test and control lines appearing, a fourth pair of point coordinates (blue dotted point 5) was recorded and the distance was calculated between points 2 and 5. This allowed the location of the remaining coordinates (red dotted points 6, 7, and 8) to be determined based on the translation of points 1, 3, and 4 (Fig. 1E). Each image was then manually annotated with three or four recorded points, and binary labels were created where test/control line regions were labeled as 1, and all other areas were labeled as 0. CRISPR-Cas-based LFAs commonly produce a faint test line even when testing a negative sample that does not contain the nucleic acid target,^29^ which can lead to a false-positive test result based on visual interpretation. Therefore, all photos of LFA devices tested on negative samples, including those presenting a faint test line, were manually labeled as a single (control) line so that the ML models would classify these images as a negative test result.

#### Image preprocessing

**Fig. 2:**
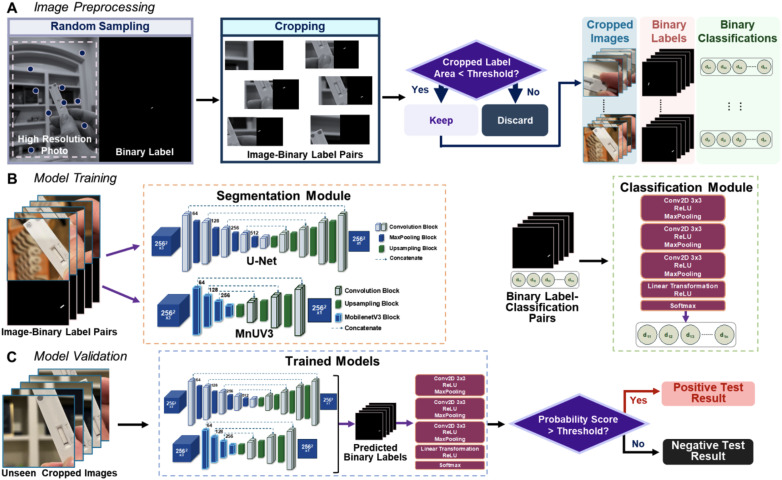
Overview of the workflows for image preprocessing, model training and model validation. (A) Image preprocessing consisted of random sampling and cropping of high-resolution LFA device photos, cropping corresponding binary labels and discarding image-binary label pairs with label areas below the threshold. (B) The segmentation module was trained by inputting cropped LFA device images and corresponding cropped human-annotated binary labels, and the classification module was trained by inputting cropped human-annotated labels and corresponding binary classification records. (C) The trained models were validated by inputting unseen cropped images and comparing the model-generated classification decisions with the ground truth.

LFA device photos and corresponding binary labels were preprocessed to facilitate the training and evaluation of the segmentation module. A schematic illustration of the workflow for preprocessing the photos and binary labels is shown in Fig. 2A. To reduce the computational load and prevent predictable patterns, high-resolution photos of LFA devices were randomly sampled and cropped into smaller images. High-resolution iPhone 13 photos (1536 × 2048 pixels) were cropped to 512 × 512 pixels and high-resolution Samsung Galaxy A52 photos (3468 × 4624 pixels) were cropped to 1156 × 1156 pixels to make the proportion ratio match. The corresponding binary labels were also cropped to obtain an image-binary label pair for each cropped image. 150 subregions were sampled from LFA device photos of a positive result and 60 subregions were sampled from LFA device photos of a negative result. During subregion sampling, we set a specific threshold for the cropped LFA device images where the image-binary label pair was discarded if the sum of pixel values in the binary label was smaller than the threshold. This criterion ensures that LFA device photos with no or extremely small amounts of test/control line(s) appearing are not sampled and excluded from the dataset. For LFA device photos of a positive result, the threshold was empirically set to 100 pixels, and for LFA device photos of a negative result, the threshold was empirically set to 70 pixels. This preprocessing procedure resulted in a total dataset of 8125 image-binary label pairs, with 4253 image-binary label pairs generated from images of LFA devices tested on negative samples and 3872 image-binary label pairs generated from images of devices tested on positive samples. The total dataset was split into a 50/20/30 ratio for training and evaluating the models and validating the trained models (Fig. S2†). The training dataset was comprised of 3980 image-binary label pairs (1852 negatives and 2128 positives) and the evaluation dataset was comprised of 1707 image-binary label pairs (846 negatives and 861 positives). The validation dataset was comprised of 3146 image-binary label pairs (1569 negatives and 1577 positives) and was unseen during the training sessions and used only to validate the trained models. The cropped images captured using the Samsung Galaxy A52 smartphone were only included in the validation dataset.

#### Binary classification records

The number of connected components (clusters of white pixels in a black and white image) in the binary labels generated from the cropped images during image preprocessing was calculated using the *bwconncomp* function in MATLAB. This function identifies and records the number of connected components and their locations within an image. The results were recorded in a spreadsheet, where a binary label with two connected components was classified as 1 and a binary label with one connected component was classified as 0. Since the image-binary label pairs were only used to train and evaluate the models, binary classification records and corresponding binary labels were not generated for high-resolution LFA device photos.

#### Image augmentation

Cropped images were augmented to further diversify the dataset in order to generate more robust models. When training the segmentation module, a variety of methods were used to augment the images. Horizontal and vertical flips of images and rotation of images within 10° with respect to the horizontal and vertical directions were performed with a 50% probability of occurrence. The image quality was also degraded using various methods, including random brightness and contrast changes, defocusing (adding Gaussian blur), downscaling, and adding Gaussian noise, each having a 50% probability of occurrence. All of the image augmentation methods were performed using the open source *Albumentations* library.^30^ Empirical limits were set to prevent excessive image degradation. Brightness and contrast changes were set to be <20%, which was the default setting. Gaussian blur parameters were also set to the default setting (3 pixels < *r*_glur_ < 10 pixels, 0.1 < *σ*_gblur_ < 0.5). Downscaling scales were set between 0.3 and 0.55, and the Gaussian noise was set to a variance of up to 0.4. Augmented images were resized to 256 × 256 pixels and converted to tensor data before segmentation module training. During model training and evaluation, we investigated three different cases involving the use of degraded images: 1) degraded images were incorporated into the training dataset only, 2) degraded images were incorporated into both the training and evaluation datasets, and 3) degraded images were not incorporated in the datasets. When training the classification module, human-annotated binary labels were transformed with horizontal and vertical flips, and horizontal and vertical rotations within 10°. Each of these transformations had a 50% probability of occurrence.

#### Model training, evaluation and validation

The segmentation and classification modules were trained separately, as illustrated in Fig. 2B. The segmentation module was trained by inputting cropped LFA device images and corresponding cropped human-annotated binary labels. The classification module was trained by inputting cropped human-annotated labels and corresponding binary classification records. The classification module outputted probability scores ranging from 0 to 1, which were subsequently converted into binary classification decisions based on a threshold value. Corresponding pairs of predicted probability scores and ground truth binary classification records were used to generate receiver operating characteristic (ROC) curves, and the closest point to coordinate (0, 1) of the ROC space and its corresponding threshold was determined for transforming probability scores to binary classifications. Probability scores above this threshold value were classified as a positive result and probability scores below the threshold value were classified as a negative result. For all training sessions, ten trials were run for each model using different combinations of datasets with and without degraded images. Consistent hyperparameters were used to train the segmentation modules with 300 epochs, a batch size of 16, and a learning rate of 0.0001 with the Adam optimizer. The classification module was trained with the ClassNet architecture with 300 epochs, a batch size of 128, and a two-step learning rate with the Adam optimizer. Within the first 20 epochs, the learning rate was set to 0.001 and 0.0001 thereafter.

Various metrics were used to evaluate the performance of the segmentation module during the training process. Binary cross entropy (BCE) loss^31^ was used to assess the segmentation loss:
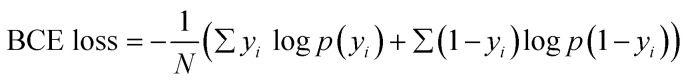
where *y*_*i*_ is the binary classification (0 or 1), *p* is the probability of *y*_*i*_ and *N* is the size of the dataset.

The Dice score^32^ was used to assess the segmentation accuracy:
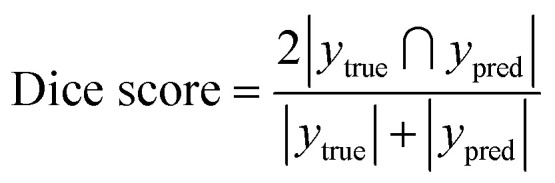
where *y*_true_ is the set of pixel values of the human-annotated labels and *y*_pred_ is the set of pixel values of the model-predicted labels.

The performance of the classification module was assessed using the classification accuracy:
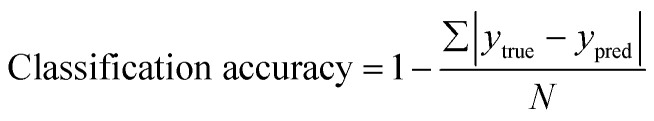


To validate the functionality of the trained models, unseen cropped images from the validation dataset were fed into the models (Fig. 2C). When evaluating the performance of the models, the predicted classification decisions were compared with the ground truth, and the results were classified into four groups: true positive (TP), true negative (TN), false positive (FP), and false negative (FN). Three metrics were used to assess the model's performance: accuracy, sensitivity and specificity, which were calculated using the following equations:
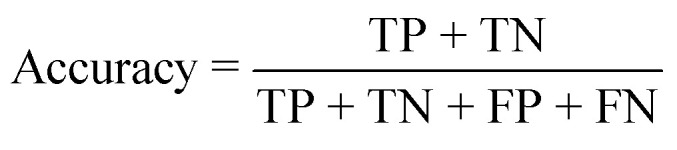

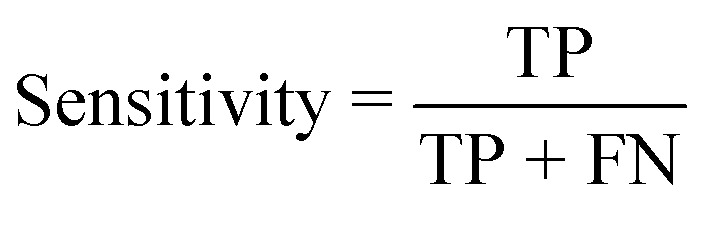

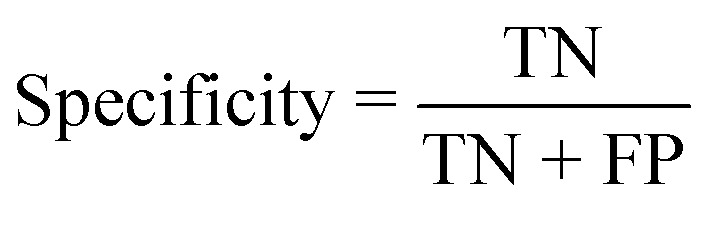


All computational experiments and analyses were conducted on a Linux-based computing server with two AMD Ryzen Threadripper PRO 3975WX 32-Core CPUs and four NVIDIA GeForce RTX 4090 Ti GPUs. Each individual experiment required one GPU at a time, though multiple GPUs were available. MATLAB 2023a was used to curate the dataset and manually annotate the binary labels. All subsequent investigations and analyses were conducted using Python 3.11.0 with Visual Studio Code 1.82. A list of Python packages used in this work is presented in Table S2.† All deep learning network-based experiments were implemented with the open-source Pytorch library using CUDA version 11.5.

## 3. Results and discussion

### 3.1 Evaluation of cropped images and human-annotated binary labels

In our approach, LFA device photos and corresponding binary labels were cropped into smaller images to reduce the computational load. To evaluate the suitability of using cropped images and binary labels, we assessed the distribution of the average image intensity from a dataset of 355 high-resolution photos and 8125 cropped images as well as the distribution of the average labeled area of binary labels corresponding to the high-resolution photos and cropped images. These distributions were transformed into a density plot where the total area under each curve was set equal to one. We also estimated the probability density distribution by calculating the kernel density function to better identify the peak location and overall pattern of the distribution of the data.^33^

The average image intensity of the high-resolution photos (positives and negatives) was ∼120 with a normal distribution between 0 and 200 [the image intensity ranges from 0 (black) to 255 (white)], indicating that most of the images in this dataset had optimal lighting (Fig. S3A†). The average image intensity of the cropped images was marginally higher (∼140) with a slightly wider normal distribution ranging from 0 to 240. Thus, cropping the high-resolution photos helped to remove extremely low and high-intensity pixels corresponding to dark (*e.g.*, shadows) or overly bright regions in the photos. Additionally, test/control lines appeared in all of the cropped images due to the binary label pixel area threshold criterion that was implemented during image preprocessing. We also examined the distribution of the average labeled area density in human-annotated binary labels corresponding to high-resolution photos and cropped images. The average labeled area in the binary labels corresponding to high-resolution photos of LFA devices tested on positive samples ranged from 1 to 2829 pixels and the average labeled area in the binary labels corresponding to photos of LFA devices tested on negative samples ranged from 1 to 1984 pixels (Fig. S3B†). The distribution of the average labeled area in the binary labels corresponding to the cropped images from positive and negative samples was narrower, ranging from 1 to 2139 pixels and 1 to 1620 pixels, respectively.

### 3.2 Segmentation performance of the ML models

We first evaluated the accuracy and loss of the U-Net- and MnUV3-based models using the training and evaluation datasets with and without degraded images. For both models, the accuracy rapidly increased within 50 epochs, with the maximum accuracy being achieved at ∼290–300 epochs (Fig. S4†). The inclusion of degraded images in the training and evaluation datasets for U-Net- and MnUV3-based models resulted in a 6.8% and 8.3% drop, respectively, in the training accuracy compared to the use of datasets without degraded images. The mean and max accuracies of both models at the best performing epoch using different combinations of datasets are summarized in Table S3.† The U-Net- and MnUV3-based models both offered high segmentation performance with a mean accuracy ranging from 90.1–97.2% and 88.8–97.2% using the training dataset and 88.7–92.9% and 87.8–96.1% using the evaluation dataset, respectively, regardless of whether degraded images were included in the datasets. Similarly, the U-Net- and MnUV3-based models exhibited low losses (using datasets with and without degraded images). These results indicate that the U-Net- and MnUV3-based models offer a similar learning ability during the training process and a similar ability to identify the test/control line(s) in LFA device images using the evaluation dataset. Furthermore, both models performed exceptionally well in identifying the test/control line(s) when the training and evaluation datasets included degraded images.

Next, we evaluated the segmentation performance of the trained models using the validation dataset. We assessed the segmentation accuracy by calculating the Dice score between the model-predicted binary labels and human-annotated binary labels based on the ground truth. We first studied the relationship between the amount of the test/control line(s) appearing in the cropped images and the segmentation accuracy, quantified by the Dice score, using datasets with and without image degradation. The U-Net- and MnUV3-based models resulted in more clustered and overall higher Dice scores, where >95% of the scores exceeded 0.9 when the training dataset did not include degraded images (Fig. S5A†). Among the three different cases where image degradation was used during the model training, >95% of the tests resulted in Dice scores ≥0.8. The majority of the tests resulting in low (<0.6) Dice scores are due to very small amounts of the test/control line(s) appearing in the image (corresponding to a labeled area <100 pixels in the binary labels). We also evaluated the segmentation performance of the models in analyzing images of LFA devices tested on positive *vs.* negative samples. For both models, a wider Dice score distribution was observed when analyzing images of devices tested on negative samples compared to those tested on positive samples (Fig. S5B†). Additionally, by using datasets with and without image degradation, the U-Net- and MnUV3-based models performed similarly where the lowest Dice scores were >0.4 when compared to the ground truth.

**Fig. 3:**
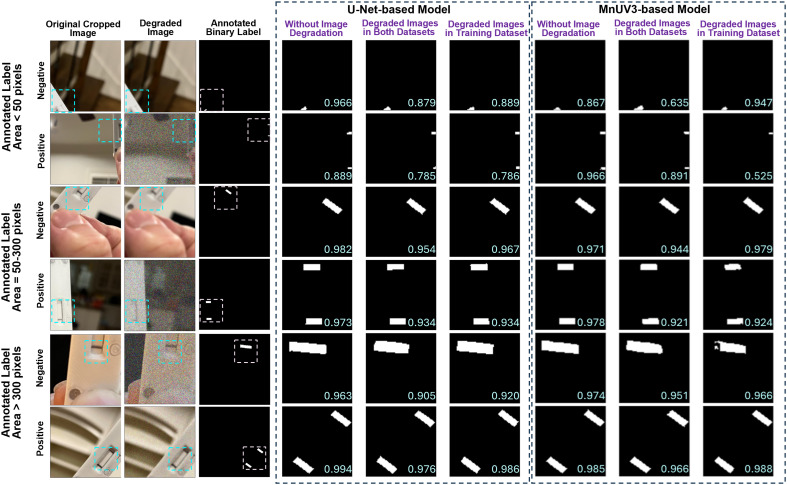
Segmentation performance of the ML models based on the amount of test/control lines appearing in the cropped image. Presented cropped and degraded images, corresponding human-annotated binary labels and model-predicted binary labels generated by the U-Net- and MnUV3-based models for images of LFA devices tested on positive and negative samples. Dice scores (calculated from the human-annotated binary labels and the model-predicted binary labels) are located in the lower right corner of model-predicted binary labels.

We further analyzed the segmentation performance by testing the trained models using the validation dataset and comparing the model-predicted binary labels with the human-annotated binary labels for different amounts of the test/control line(s) appearing in the cropped image (label area < 50 pixels, 50 ≤ label area ≤ 300 pixels, or label area > 300 pixels). The MnUV3-based model identified the test/control line(s) with high accuracy (Dice scores > 0.9) when compared to human-annotated binary labels when analyzing cropped LFA device images with corresponding binary label areas ≥ 50 pixels (Fig. 3). However, for images with corresponding binary label areas < 50 pixels, the MnUV3-based model exhibited significantly lower segmentation performance (Dice scores = ∼0.6–0.8) compared to the human-annotated binary labels, either producing inaccurate segmentation or identifying only one line even when both the test and control lines appeared in the image. Additionally, incorporating image degradation in both the training and evaluation datasets or only in the training dataset during the model training did not significantly improve the segmentation performance, as indicated by similarities in the Dice score for datasets with and without degraded images.

We assessed the relationship between the labeled area in the model-predicted binary label to the labeled area in the human-annotated binary labels by plotting the labeled area determined by both methods in a scatter plot and performing linear regression analysis. The results of this analysis showed that the labeled area predicted by the models were highly correlated (*R*^2^ = 0.86–0.96) with those determined by human annotations for labeled areas from 0 to 300 pixels (Fig. S6†). Additionally, Bland–Altman analysis was performed on this data, which revealed that there was minimal bias (<2.5%), expressed as the percentage difference between the model-predicted binary label areas and the human-annotated binary label areas for the U-Net- and MnUV3-based models (Fig. S7†).

### 3.3 Performance of the classification module

We assessed the performance of the classification module by analyzing the accuracy and loss from 0 to 300 epochs. The model achieved >95% mean accuracy during model training and evaluation within the first 50 epochs (Fig. S8†). The highest performance was achieved at epoch 294 where the model achieved a mean accuracy of 96.7% (Table S4†) with mean losses of 5.6% and 5.9% during training and evaluation, respectively. The close agreement between the training and evaluation accuracy/loss curves indicates that the model generalized well to new data.

**Fig. 4:**
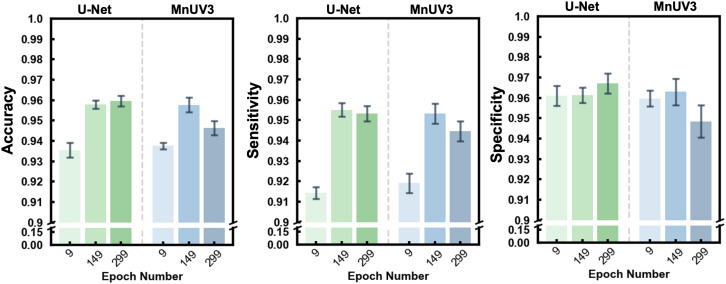
Classification performance of the ML models using the optimal classification threshold values. Plots of accuracy, sensitivity and specificity for the U-Net- and MnUV3-based models at epochs 9, 149 and 299. Each bar represents the mean ± standard deviation of ten trials.

Studies were performed to determine the optimal probability score threshold value used to generate binary classification decisions. Since the use of datasets with degraded images did not significantly improve the segmentation performance of the model, these studies were performed using training datasets that did not include degraded images. Given the ability of the classification module to converge quickly (within 50 epochs), we evaluated threshold values at three different epochs (9, 149, and 299). The classification module was tested by inputting model-predicted binary labels generated from the validation dataset using the U-Net- and MnUV3-based models. The optimal threshold value was determined by calculating the nearest point to coordinate (0, 1) in the ROC curves (representing 100% sensitivity and 100% specificity), which was selected as the optimal value for that specific trial. The optimal threshold values are presented in Table S5.† We assessed three different performance metrics (accuracy, sensitivity and specificity) of the classification module using the optimal classification threshold values at epochs 9, 129 and 299. For this analysis, we selected the parameters that yielded the highest segmentation accuracy for each model. The accuracy and sensitivity of both models at epoch 9 was significantly lower than those at epochs 149 and 299 (Fig. 4). Excellent classification performance was achieved with both the U-Net and MnUV3-based models, with mean accuracies of 96.0% and 95.9%, mean sensitivities of 95.5% and 95.2% and mean specificities of 96.8% and 96.3%, respectively.

### 3.4 Validation of the ML models

**Fig. 5:**
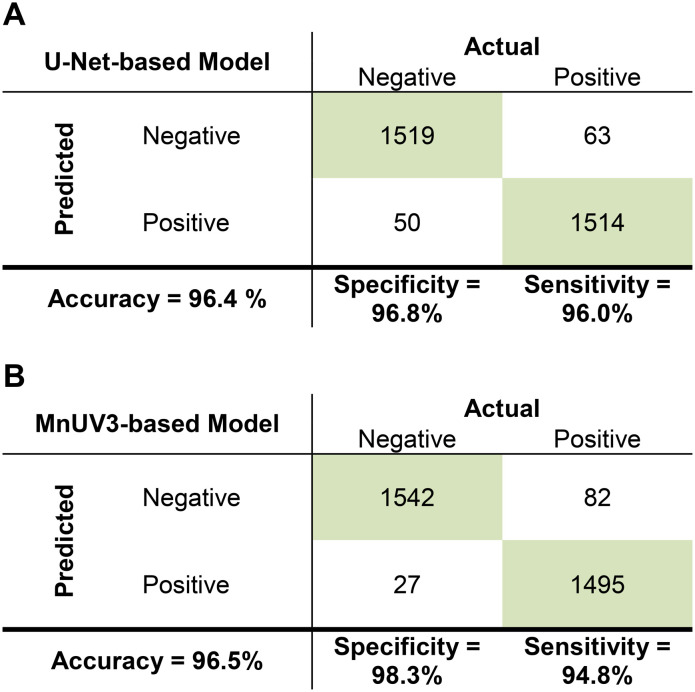
Confusion matrices showing the performance of the ML models in analyzing images of tested CRISPR-Cas13-based LFA devices for the detection of the SARS-CoV-2 N gene. The results from one trial for the (A) U-Net-based model and (B) MnUV3-based model are presented.

**Fig. 6:**
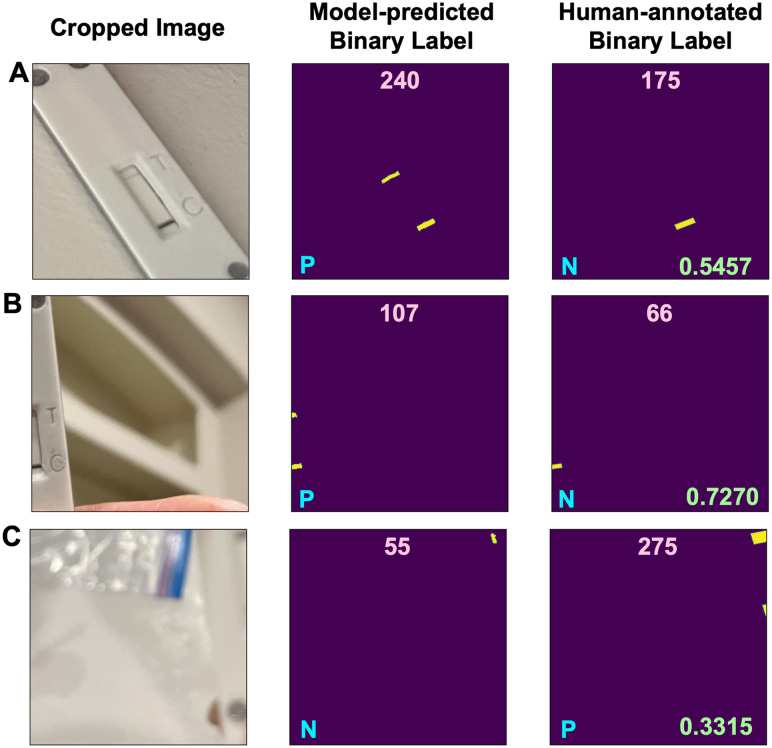
Representative cropped images, corresponding model-predicted binary labels and human-annotated binary labels for incorrect outcomes. (A) Extra pixels generated in the binary label during segmentation. Missing pixels in the binary label generated during segmentation due to (B) the test/control line(s) being too close to the edge of the image or (C) the image being too blurry. The label area (pixels) is indicated at the top of the binary labels, the classification decision is indicated at the bottom left corners of the binary labels and the Dice score is indicated at the bottom right corners of the human-annotated labels.

We validated the trained U-Net- and MnUV3-based models by testing 3146 unseen cropped images (1569 negative, 1577 positive). Both the U-Net- and MnUV3-based models classified the images with excellent accuracy (96.4% and 96.5%, respectively), sensitivity (96.0% and 94.8%, respectively), and specificity (96.8% and 98.3%, respectively) based on the ground truth (Fig. 5). The performance of these models is comparable to what was reported in prior studies employing ML for the interpretation of smartphone-captured images of LFA test results while our method does not require the images to be standardized (Table S6†). To identify sources of error and better understand the limitations of these models, we analyzed several cases where the models generated incorrect outcomes. One source of error involved instances where extra pixels were generated in the binary label during segmentation (*e.g.*, a faint test line was incorrectly classified as a positive result) (Fig. 6A). Another source of error involved instances of missing pixels in the binary label generated during segmentation due to the test/control line(s) being too close to the edge of the image (Fig. 6B) or if the image was too blurry (Fig. 6C). Improvements in the model training process and/or the development of more robust algorithms would help to resolve these issues and increase the accuracy of the models. In this work, true positives were generated by testing samples spiked with the SARS-CoV-2 N gene at concentrations ranging from 1 nM to 100 nM, where 1 nM was the lower limit of detection (LOD) of the assay. Based on this LOD, the models were trained to distinguish faint test lines generated for positive samples containing the SARS-CoV-2 N gene at concentrations as low as 1 nM from faint test lines generated for true negative samples. Samples containing the SARS-CoV-2 N gene at concentrations lower than 1 nM could potentially produce a faint test line, resulting in a false negative result. To resolve this issue, optimization of the CRISPR-Cas assay can be performed to enhance its analytical sensitivity, which would result in a lower LOD and reduce the likelihood of a false negative result.

## 4. Conclusion

We have developed two ML algorithms (one based on U-Net and the other based on MnUV3) for rapid and automated interpretation of CRISPR-Cas-based LFA test results. Using smartphone-captured images of LFA devices tested on samples spiked with various concentrations of the SARS-CoV-2 N gene, we demonstrate the ability of these models to classify the results as positive or negative within 0.2 s with 96.5% accuracy. This high accuracy was achieved using images of LFA devices captured in various orientations, under different lighting conditions, and with different backgrounds and image qualities, demonstrating the robustness of these ML models and their potential to be used in real-world environments. All of the images used in this work were captured indoors and additional studies using images captured outdoors would be useful in determining the capability of these models to operate in less controlled environments. One potential limitation of this work was the use of only two different smartphone models (iPhone 13 and Samsung Galaxy A52) for capturing images of LFA devices and further studies are needed to evaluate the performance of the ML models using other smartphone models with different cameras. While this work was focused on interpreting test results of a CRISPR-Cas-based LFA for the detection of the SARS-CoV-2 N gene, we envision that these models can be adapted for other CRISPR-Cas-based LFAs designed to detect other nucleic acid targets or other LFA formats by retraining them with photos of these types of LFA devices and formats. These efforts would expand the versatility of these models and advance progress in the use of ML to interpret CRISPR-Cas-based diagnostic tools for self- and at-home testing.

## Data availability

The data supporting this article have been included as part of the ESI.† Further data are available upon reasonable request from the authors. The custom code used in this study is available in this GitHub repository: https://github.com/cc0231/Lateral-Flow-Interpretation-Code/.

## Author contributions

Conceptualization: M. X., P. L.; data curation: M. X., D. G.; formal analysis: M. X., D. G.; funding acquisition: P. L.; investigation: M. X.; methodology: M. X., D. G.; project administration: P. L., X. G.; resources: M. X., D. G., E. O.; software: M. X.; supervision: P. L., X. G.; validation: M. X.; visualization: M. X., D. G.; writing – original draft: D. G., M. X., E. O., P. L.; writing – review & editing: D. G., M. X., E. O., P. L., X. G. All authors have read and agreed to the published version of the manuscript.

## Conflicts of interest

The authors declare no conflicts of interest.

## Supplementary Material

SD-004-D4SD00314D-s001
