# Supplementary material for: Rapid and automated interpretation of CRISPR-Cas13-based lateral flow assay test results using machine learning
Source: Sens Diagn. 2024 Dec 26;4(2):171–81. doi: 10.1039/d4sd00314d (PMC11726308; doi:10.1039/d4sd00314d)
Supplement: SD-004-D4SD00314D-s001 [file SD-004-D4SD00314D-s001.pdf]

Supplementary Information (SI) for Sensors & Diagnostics.
This journal is © The Royal Society of Chemistry 2024

Supplementary Information for

# Rapid and automated interpretation of CRISPR-Cas13-based lateral flow assay test results using machine learning

Mengyuan Xue,†[a] Diego H. Gonzalez,†[a] Emmanuel Osikpa,[b] Xue Gao,[b,c,d,e] and Peter B. Lillehoj[*a,f]

[a] Department of Bioengineering, Rice University, Houston, TX, 77030, USA

[b] Department of Chemical and Biomolecular Engineering, Rice University, Houston, TX 77005, USA

[c] Department of Chemical and Biomolecular Engineering, University of Pennsylvania, Philadelphia, PA 19104, USA

[d] Department of Bioengineering, University of Pennsylvania, Philadelphia, PA 19104, USA

[e] Center for Precision Engineering for Health, University of Pennsylvania, Philadelphia, PA 19104, USA

[f] Department of Mechanical Engineering, Rice University, Houston, TX, 77005, USA

*Corresponding author: lillehoj@rice.edu

**Table S1.** Sequences of crRNA, SARS-CoV-2 N gene target and LFA reporter.

| Name | Sequence (5’ -> 3’) | Source | Note | Ref. |
|---|---|---|---|---|
| **crRNA** | | | | |
| SARS-CoV-2 crRNA | GACUACCCCAAAAACGA AGGGGACUAAAACaaucug aggguccaccaaacguaaugcg | IDT | - | 7 |
| **Reporter** | | | | |
| LFA Reporter | /FAM/rUrU rUrUrU rUrUrU rUrUrU rUrUrU /Bio/ | IDT | - | 7 |
| **Target** | | | | |
| SARS-CoV-2 Target | gucugauaauggaccccaaaaucagcgaaa ugcaccccgcauuacguuugguggacccu cagauucaacuggcaguaaccagaauggag aacgcaguggggcgcg | IDT | SARS-CoV-2 (Ref Seq: NC_045512.2) N gene fragment: 28,276–28,380 | 7 |

**Table S2.** Summary of Python packages and corresponding version used in this work.

| Package | Version |
|---|---|
| albumentation | 1.4.6 |
| efficientnet | 0.7.1 |
| imageio | 2.34.1 |
| matplotlib | 3.8.4 |
| numpy | 1.26.4 |
| opencv-python | 4.9.0.80 |
| pandas | 2.2.2 |
| pillow | 10.3.0 |
| scikit-image | 0.23.2 |
| scikit-learn | 1.4.2 |
| scipy | 1.13.0 |
| SimpleITK | 2.3.1 |
| torchsummary | 1.5.1 |
| torchtoolbox | 0.1.8.2 |
| torchvision | 0.18.0 |
| torch | 2.3.0 |

**Table S3.** Accuracy of the U-Net- and MnUV3-based models tested using the training and evaluation datasets. The models were trained using the following hyperparameters: Learning rate, 1e-4; Optimizer, Adam; Batch size, 16; Number of epochs trained, 300.

| Architecture | Image Degradation | | Results Using Training Dataset | | Results Using Evaluation Dataset | | |
|---|---|---|---|---|---|---|---|
| | Training | Evaluation | Mean Training Accuracy | Max Training Accuracy | Mean Validation Accuracy | Max Validation Accuracy | Epoch # |
| **U-Net** | N | N | $0.972 \pm 0.001$ | $0.974 \pm 0.0004$ | $0.958 \pm 0.002$ | $0.961 \pm 0.001$ | 287 |
| **U-Net** | Y | Y | $0.904 \pm 0.002$ | $0.909 \pm 0.001$ | $0.887 \pm 0.002$ | $0.891 \pm 0.001$ | 300 |
| **U-Net** | Y | N | $0.905 \pm 0.002$ | $0.910 \pm 0.001$ | $0.929 \pm 0.002$ | $0.932 \pm 0.001$ | 298 |
| **MnUV3** | N | N | $0.972 \pm 0.002$ | $0.974 \pm 0.001$ | $0.961 \pm 0.001$ | $0.963 \pm 0.001$ | 290 |
| **MnUV3** | Y | Y | $0.889 \pm 0.002$ | $0.894 \pm 0.002$ | $0.878 \pm 0.003$ | $0.886 \pm 0.003$ | 293 |
| **MnUV3** | Y | N | $0.888 \pm 0.002$ | $0.891 \pm 0.001$ | $0.927 \pm 0.001$ | $0.930 \pm 0.001$ | 297 |

**Table S4.** Performance of the classification module tested using the training and evaluation datasets. The model was trained using the following hyperparameters: Learning rate, 1e-3 (Epoch < 20), else 1e-4; Optimizer, Adam; Batch size, 128; Number of epochs trained, 300.

| Architecture | Results Using Training Dataset | | Results Using Evaluation Dataset | | |
|---|---|---|---|---|---|
| | Mean Training Accuracy | Max Training Accuracy | Mean Validation Accuracy | Max Validation Accuracy | Epoch # |
| ClassNet | $0.968 \pm 0.005$ | $0.976 \pm 0.003$ | $0.966 \pm 0.003$ | $0.974 \pm 0.003$ | 294 |

**Table S5.** Optimal classification threshold values using the U-Net- and MnUV3-based models at epochs 9, 149 and 299. Each value represents the average from 10 trials.

| | **U-Net** | **MnUV3** |
|---|---|---|
| **Epoch 9** | 0.936 | 0.938 |
| **Epoch 149** | 0.918 | 0.922 |
| **Epoch 299** | 0.922 | 0.914 |

**Table S6.** Comparison of ML methods for interpretation of smartphone-captured images of LFA test results.

| Type of Assay | Method/ Algorithm(s) | Smartphone(s) Tested | Standardized Images[1] (Y/N) | Accuracy (%) | Sensitivity (%) | Specificity (%) | Ref. |
|---|---|---|---|---|---|---|---|
| IA | Feature extraction with self-supervised learning (FeatureNet); Model adaptation with supervised contrastive learning | Training: iPhone X Evaluation: iPhone X, iPhone 7, and Samsung Galaxy J3 | N | >99 | 98.2 ± 0.8 | 99.1± 0.2 | 1 |
| IA | Convolutional and multiscale network for LFA identification; GAN for abnormal features identification | Various iPhones and Android-based phones | Y | 98.6 | 100 | 99.28 | 2 |
| IA | SVM/KNN/Weighted-KNN/Decision-tree based classification | OnePlus One | Y | 95.56 | - | - | 3 |
| IA | Three CNNs (ResNet50, MobileNetv2, MobileNetv3) and SVM-based classification | Samsung SM-P585 tablet | Y | 80-97 | 97.8 | 100 | 4 |
| IA | Supervised ANN-based classification | Apple iPhone 4, 4S and 5 | Y | 96 | - | - | 5 |
| IA | SVM-based classification | Samsung Galaxy S7 Edge | Y | 98 | - | - | 6 |
| CRISPR-Cas | Two CNNs for segmentation; ClassNet (Convolution+Maxpolling layers) network for classification. | iPhone 13 and Samsung A52 5G | N | 96.5 | 96.0 | 98.3 | This work |

**Notes and abbreviations:**

[1] Smartphone-captured images were mathematically transformed so that all of the images in the dataset had a consistent intensity or other work-specific features.

IA - Immunoassay
GAN - Generative adversarial network
SVM - Support vector machine
KNN - K-nearest neighbors
CNN - Convolutional neural network
ANN - Artificial neural network

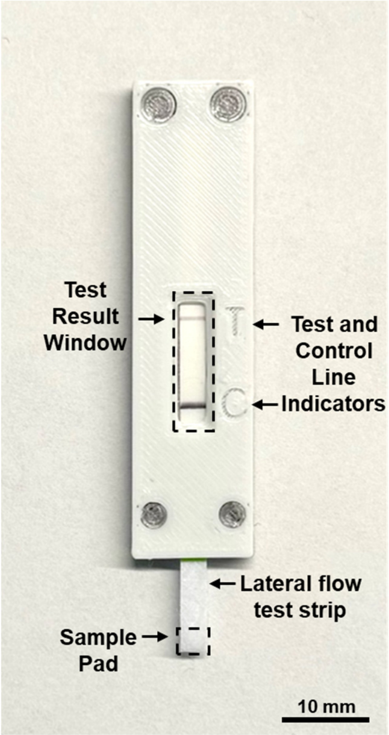


**Figure S1.** Photograph of the LFA device. Scale bar, 10 mm.

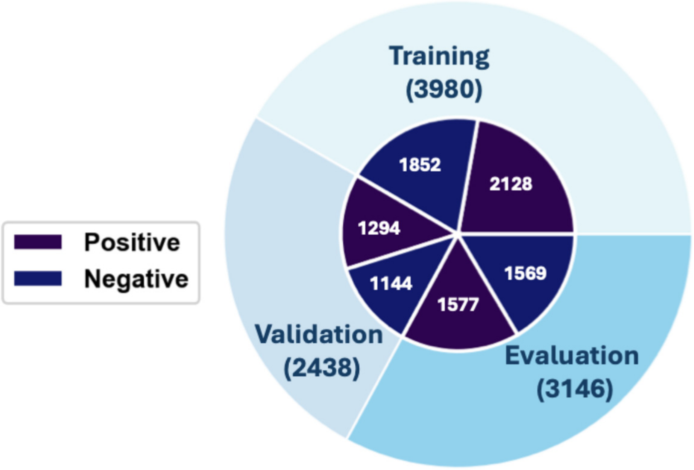


**Figure S2.** Composition of the dataset. The outer ring illustrates the distribution of images in the training, evaluation and validation datasets and the inner ring illustrates the distribution of positives and negatives within these datasets.

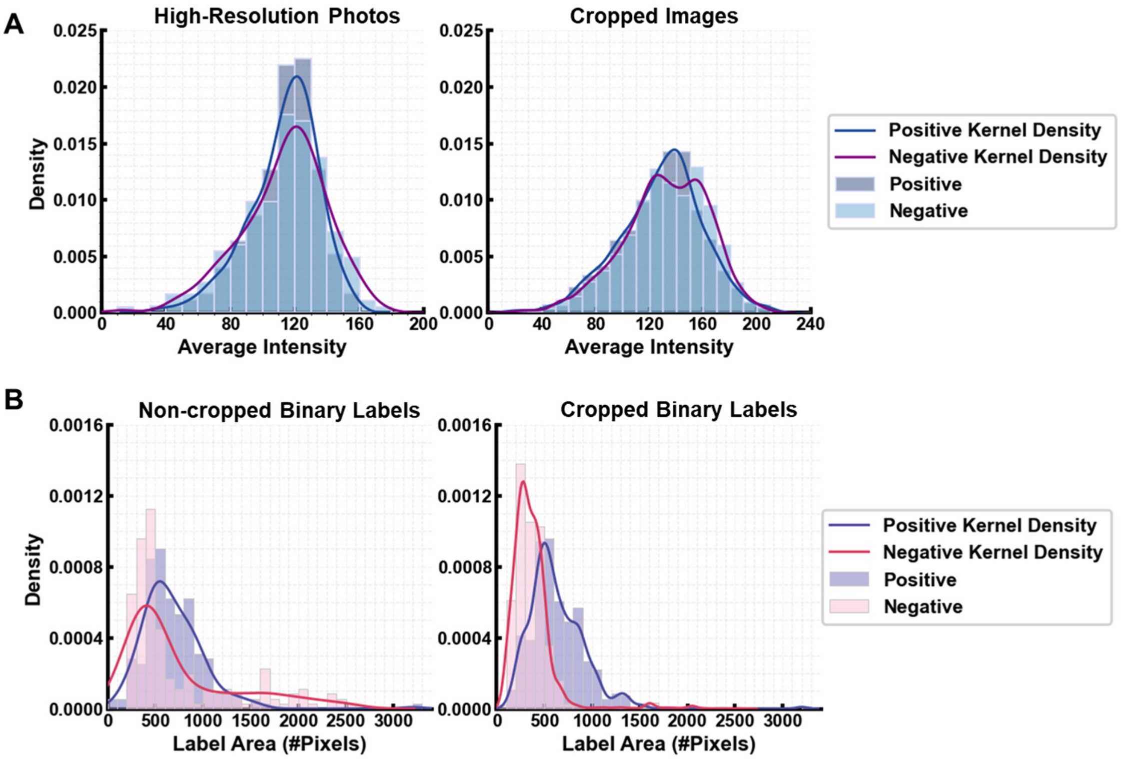


**Figure S3.** (A) Density plots of average image intensity distribution for high-resolution photos and cropped images. (B) Density plots of average binary label area corresponding to high-resolution photos and cropped images. The distributions are normalized to 1 (area under each curve).

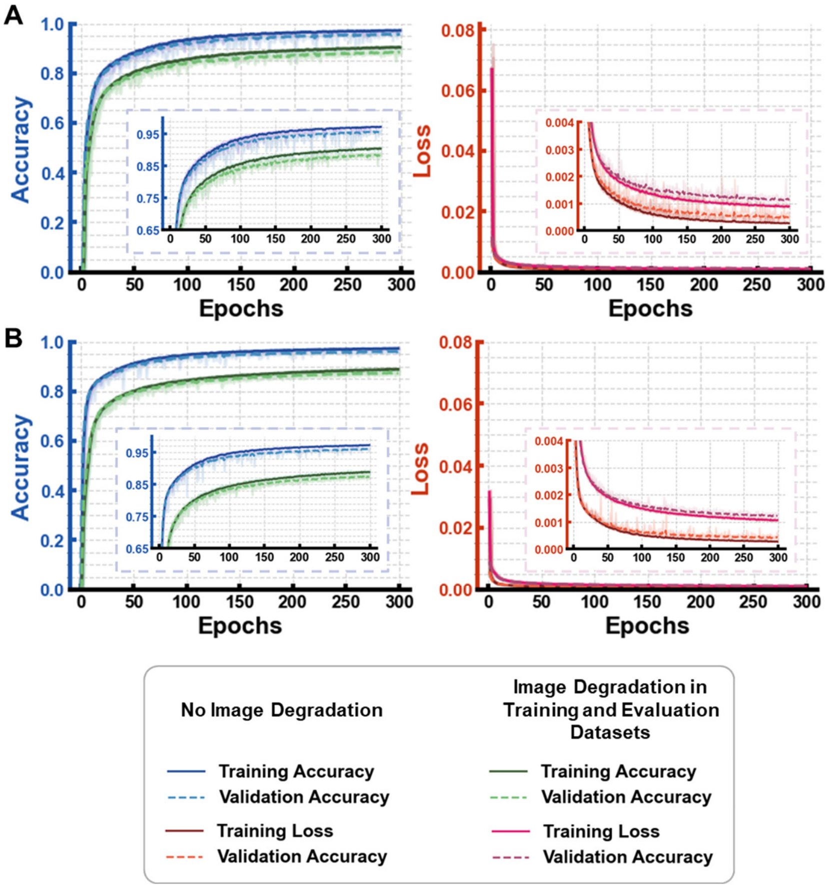


**Figure S4.** Segmentation performance of ML models. Accuracy and loss vs. epoch curves for the (A) U-Net-based model and (B) MnUV3-based model. Subplots show magnified views of the curves at upper (accuracy: 0.65-1.0) and lower (loss: 0.0-0.004) y-axis bounds. Each curve represents the mean of 10 trials.

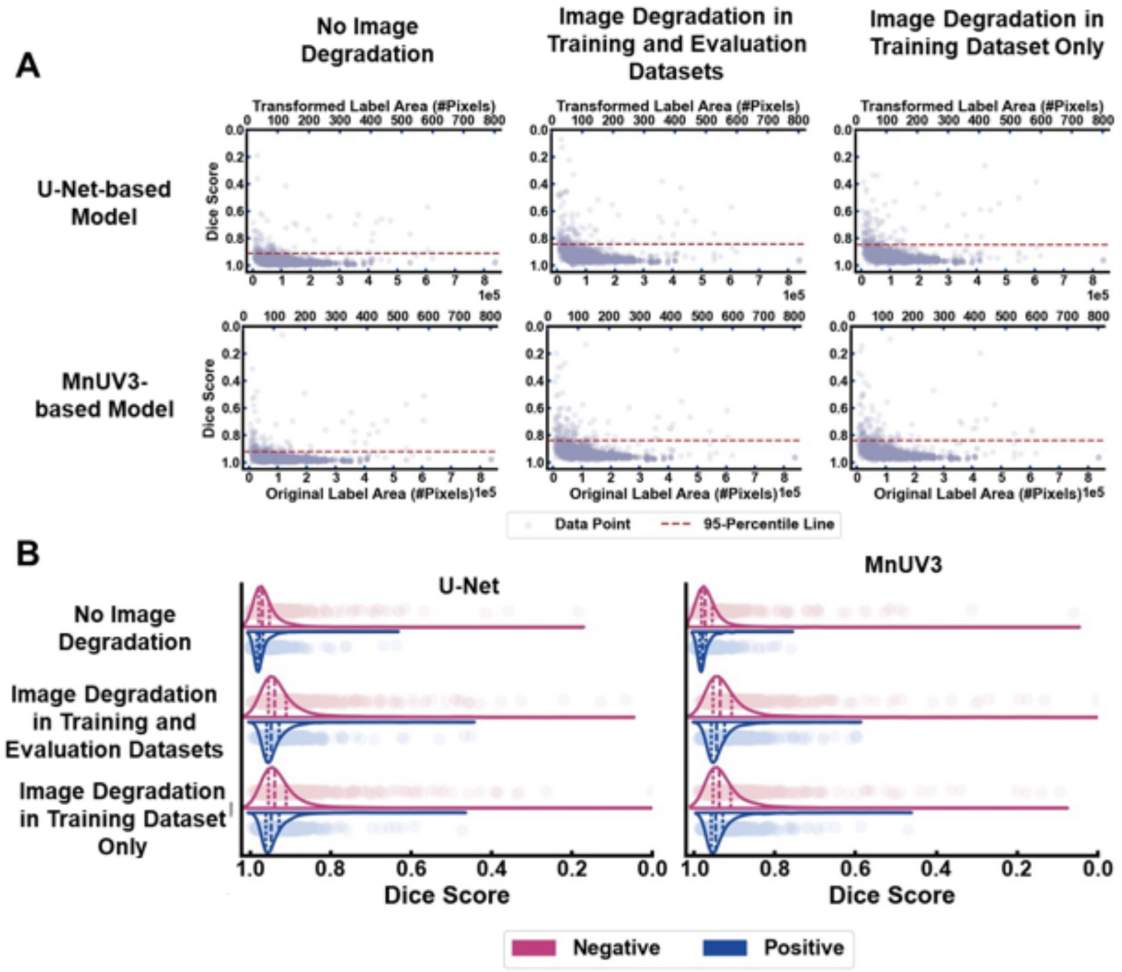


**Figure S5.** Segmentation performance of the trained ML models. (A) Dice score scatter plot vs label area (transformed and original) for the U-Net- and MnUV3-based models using different datasets with and without image degradation. The horizontal dashed lines represent the boundary of the 95$^{th}$ percentile. (B) Dice score distribution violin plot categorized by images of LFA devices tested on negative and positive samples.

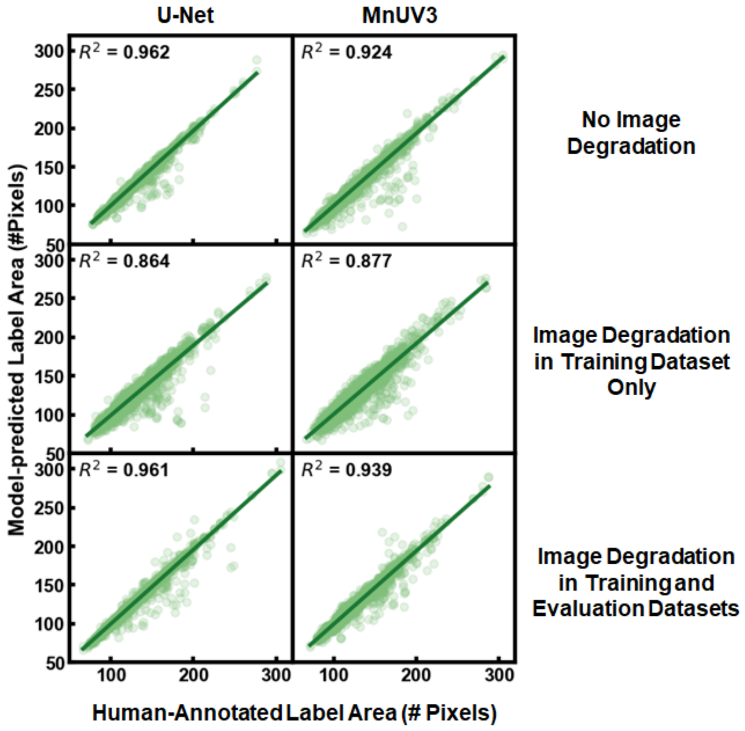


**Figure S6.** Comparison of binary label areas generated from cropped LFA device images predicted by the U-Net- and MnUV3-based models to those determined by human annotation.

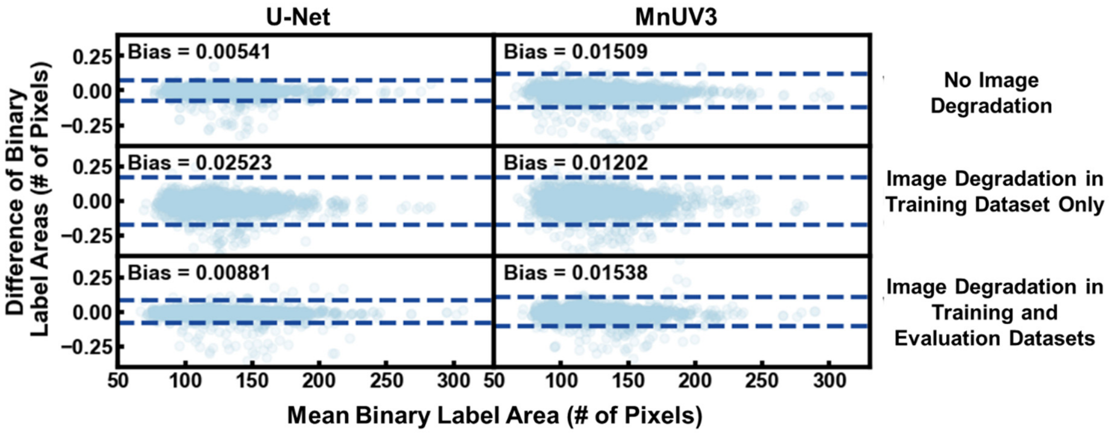


**Figure S7.** Bland-Altman plots (model-predicted binary label area – human-annotated binary label area vs. mean binary label area) showing the agreement between the model-predicted binary label areas and human-annotated binary label areas generated from cropped LFA device images.

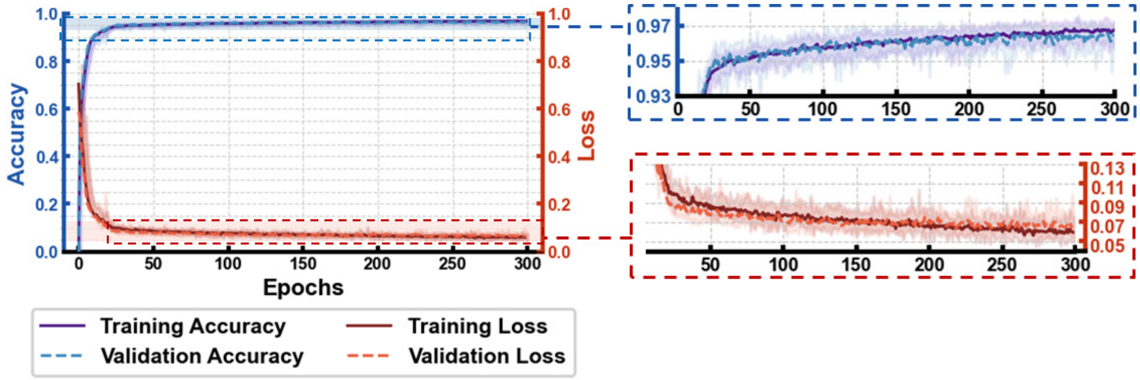


**Figure S8.** Performance of the classification module. Accuracy and loss vs. epoch curves for model training and evaluation. Insets show magnified views of the curves at upper (accuracy: 0.93-0.98) and lower (loss: 0-0.13) y-axis bounds. Each curve represents the mean of 10 trials.

## References


1. S. Arumugam, J. Ma, U. Macar, G. Han, K. McAulay, D. Ingram, A. Ying, H. H. Chellani, T. Chern, K. Reilly, D. A. M. Colburn, R. Stanciu, C. Duffy, A. Williams, T. Grys, S.-F. .Chang and S. K. Sia, *Commun. Med.*, 2023, **3**, 1–14.
2. A. D. Beggs, C. C. S. Caiado, M. Branigan, P. Lewis-Borman, N. Patel, T. Fowler, A. Dijkstra, P. Chudzik, P. Yousefi, A. Javer, B. Van Meurs, L. Tarassenko, B. Irving, C. Whalley, N. Lal, H. Robbins, E. Leung, L. Lee and R. Banathy, *Cell Rep. Med.*, 2022, **3**, 100784.
3. H. J. Min, H. A. Mina, A. J. Deering and E. Bae, *J. Microbiol. Methods*, 2021, **188**, 106288.
4. V. Turbé, C. Herbst, T. Mngomezulu, S. Meshkinfamfard, N. Dlamini, T. Mhlongo, T. Smit, V. Cherepanova, K. Shimada, J. Budd, N. Arsenov, S. Gray, D. Pillay, K. Herbst, M. Shahmanesh and R. A. McKendry, *Nat. Med.*, 2021, **27**, 1165–1170.
5. A. Carrio, C. Sampedro, J. Sanchez-Lopez, M. Pimienta and P. Campoy, *Sensors*, 2015, **15**, 29569–29593.
6. K. H. Foysal, S. E. Seo, M. J. Kim, O. S. Kwon and J. W. Chong, *Sensors*, 2019, **19**, 4812.
7. J. Yang, Y. Song, X. Deng, J. A. Vanegas, Z. You, Y. Zhang, Z. Weng, L. Avery, K. D. Dieckhaus, A. Peddi, Y. Gao, Y. Zhang and X. Gao, *Nat. Chem. Biol.*, 2023, **19**, 45–54.
